# A nuclear magnetic resonance study of water in aggrecan solutions

**DOI:** 10.1098/rsos.150705

**Published:** 2016-03-09

**Authors:** Richard J. Foster, Robin A. Damion, Thomas G. Baboolal, Stephen W. Smye, Michael E. Ries

**Affiliations:** 1 School of Physics and Astronomy, University of Leeds, Leeds LS2 9JT, UK; 2 Academic Division of Medical Physics, University of Leeds, Leeds LS2 9JT, UK; 3 Leeds Institute of Rheumatic and Musculoskeletal Medicine, University of Leeds, St James's University Hospital, Leeds LS9 7TF, UK; 4 National Institute for Health Research, Leeds Musculoskeletal Biomedical Research Unit, Chapel Allerton Hospital, University of Leeds, Leeds LS2 9LN, UK

**Keywords:** aggrecan, glycosaminoglycan, relaxation, diffusion, activation energy, chemical exchange

## Abstract

Aggrecan, a highly charged macromolecule found in articular cartilage, was investigated in aqueous salt solutions with proton nuclear magnetic resonance. The longitudinal and transverse relaxation rates were determined at two different field strengths, 9.4 T and 0.5 T, for a range of temperatures and aggrecan concentrations. The diffusion coefficients of the water molecules were also measured as a function of temperature and aggrecan concentration, using a pulsed field gradient technique at 9.4 T. Assuming an Arrhenius relationship, the activation energies for the various relaxation processes and the translational motion of the water molecules were determined from temperature dependencies as a function of aggrecan concentration in the range 0–5.3% w/w. The longitudinal relaxation rate and inverse diffusion coefficient were approximately equally dependent on concentration and only increased by upto 20% from that of the salt solution. The transverse relaxation rate at high field demonstrated greatest concentration dependence, changing by an order of magnitude across the concentration range examined. We attribute this primarily to chemical exchange. Activation energies appeared to be approximately independent of aggrecan concentration, except for that of the low-field transverse relaxation rate, which decreased with concentration.

## 1. Introduction

Aggrecan is the main proteoglycan found in cartilage [1]. The macromolecule tends to be polydisperse with an average molecular weight of approximately 2.5 MDa [2]. It has a core protein of approximately 300 kDa [3] and has of the order of 100 attached glycosaminoglycan (GAG) side chains, of which chondroitin sulfate is the most dominant (approx. 20 kDa), but also present are the shorter keratan sulfates (5–15 kDa) [1]. The GAG chains are linear polymers of approximately 30 disaccharide monomers with each disaccharide containing carboxylate and sulfate groups which are negatively charged under physiological pH conditions. Each disaccharide is doubly negatively charged and thus the highly charged GAG chains give the aggrecan molecule an extended, semi-rigid bottlebrush conformation in solution [3].

In their recent review, Chandran & Horkay [3] propose that the properties of aggrecan solutions are concentration-dependent. Four different regimes are observed; dilute and dilute-transition (less than 0.0075 g cm^−3^), assembly (0.0075–0.01 g cm^−3^), physiological (0.01–0.1 g cm^−3^) and concentrated (0.1–0.7 g cm^−3^). In the dilute range, the average separation distance between protein cores is of the same order as the aggrecan molecule diameter and the solution behaves as one of non-interacting molecules. As the concentration increases, the molecules begin to overlap and ‘microgel-like’ [3,4] clusters begin to form. At about a concentration of 0.002 g cm^−3^ (the onset of the dilute-transition region), the diffusion coefficient of the aggrecan molecule begins to decrease [5] and the reduced viscosity shows a local minimum [3,6]. At the start of the assembly range, the molecules are believed to undergo self-assembly [4], but the nature of this self-assembly is not clearly understood. At a concentration of about 0.01 g cm^−3^, we reach the lower value of the physiological range where the distance between protein cores is much smaller than the aggrecan diameter [3].

In cartilage, proteoglycans constitute approximately 30–35% of the dry weight of the tissue. They form large aggregates with hyaluronic acid and cross-link with the collagen fibres (of which type-II are the dominant form) [7]. The high charge density of the GAG chains attracts cations into the tissue and this produces an osmotic pressure which swells the cartilage with interstitial water until the collagen fibres restrain it [7]. Owing to the stiffness of the GAGs and the interaction between the proteoglycan complexes and collagen, when a load is placed upon the cartilage the flow of the fluid is resisted. This provides the tissue with a shock-absorbing property that is crucial for the normal functioning of articular cartilage.

Osteoarthritis is a degenerative joint disease which affects articular cartilage and can lead to chronic disability and pain [8]. For these reasons, among others, articular cartilage has been studied in a diverse variety of ways. One such method is via the versatile techniques of nuclear magnetic resonance (NMR) and magnetic resonance imaging (MRI). MRI has been applied in several ways in an attempt to diagnose osteoarthritis or study the structure and function of articular cartilage [9,10]. This involves measuring parameters such as the relaxation times or diffusion coefficient and relating them to the known composition, functional status or mechanical properties of cartilage. Like all biological tissues, cartilage is a complex material and parameters such as the NMR relaxation times will reflect an average of all the interactions that the water molecules experience during the measurement time.

The aim of this paper is to measure the properties of a simpler system, aggrecan in aqueous solutions, and determine how key NMR parameters (the longitudinal relaxation rate *R*_1_, the transverse relaxation rate *R*_2_ and the diffusion coefficient *D*) change with aggrecan concentration. We compare measurements of these parameters at two different field strengths to investigate the effects of chemical exchange, and at a range of temperatures to determine Arrenhius activation energies. These measurements are then interpreted in terms of water–aggrecan interactions.

The intention is that this work may help inform an approach to the detailed understanding of the properties of cartilage.

## 2. Material and methods

### 2.1. Aggrecan solutions

Approximately 34 mg of aggrecan was purchased from Sigma-Aldrich Inc. This product is extracted from bovine articular cartilage, chromatographically purified, dialysed against water and sterile-filtered prior to lyophilization. The lyophilized powder is essentially salt-free. The entire quantity of aggrecan was placed in a sterile container with approximately 600 µl of sterile-filtered salt solution (table 1). After ensuring that the resulting aggrecan solution was well mixed, 200—250 µl was extracted and placed in a 5 mm diameter NMR tube and sealed. The volume that was extracted from the original container was then replaced by approximately the same volume of salt solution to create a lower concentration in that container. This procedure was repeated to create five solutions of known aggrecan concentrations (mass of aggrecan per unit mass of total solution—table 2) in sealed NMR tubes. A sixth tube of salt solution with no aggrecan was also produced at the same time. The total mass of the aggrecan/salt solution in its container was weighed at each stage of the process, with an accuracy of ±0.2 mg. This method of sample production was used due to the limited amount of aggrecan available (total 34 mg).

**Table 1.** Ionic concentrations of salt solution. Concentrations of the ions were chosen to agree approximately with values found in cartilage [11], except for calcium. The pH was approximately 7.5.

ion	concentration (mM)
Na^+^	220
K^+^	7.2
Cl^−^	108

**Table 2.** Aggrecan solution concentration as measured with respect to solution mass, *c*_m_, and sGAG concentration measured with respect to solution volume, *c*_v_.

solution	aggrecan concentration *c*_m_ (mg mg^−1^) ± 0.003	sGAG concentration *c*_v_ (mg ml^−1^)
1	0.053	43.6 ± 3.7
2	0.034	15.5 ± 0.7
3	0.019	7.5 ± 0.3
4	0.012	6.8 ± 0.3
5	0.007	4.2 ± 0.1
6	0	0

The range of the sample concentrations produced is shown in table 2. These were made so that they approximately covered the range of biological concentrations found in articular cartilage; however, it was not possible to produce samples of a higher concentrations because the aggrecan showed strong gellation during sample preparation. The highest concentration sample made here was the first at which gellation was prevented. Post-hoc analysis showed that the longitudinal relaxation time *T*_1_, transverse relaxation time *T*_2_ and *D* values of the most dilute aggrecan solution were close to that of the pure salt solution and thus it was deemed unnecessary to produce more dilute solutions.

To confirm the concentration of each aggrecan solution, samples were subsequently measured using a Blyscan GAG assay (Biocolor Ltd, UK) according to the manufacturer's instructions. The concentrations (mass of sulfated GAGs per unit volume of total solution) are also displayed in table 2.

### 2.2. Low-field nuclear magnetic resonance

Measurements of proton NMR relaxation times *T*_1_ and *T*_2_ were performed on a bench-top MARAN (Resonance Instruments Inc., Oxford, UK) 0.5 T spectrometer (20 MHz), over a range of temperatures from approximately 8°C to 38°C. *T*_1_ was measured via an inversion-recovery sequence using 16 inversion times between approximately 20 ms and 20 s. Four signal averages were acquired, a repetition time of between 12 and 15 s was used, and no dummy scans were employed. *T*_2_ was measured via a Carr–Purcell–Meiboom–Gill (CPMG) sequence, sampling every second echo (with an echo spacing of 2 ms) over 1000–3500 echoes (depending on the sample concentration and temperature), four averages, a repetition time between 12 and 15 s and no dummy scans. All relaxation data were fitted using the Resonance Instruments software.

### 2.3. High-field nuclear magnetic resonance

Measurements of proton NMR relaxation times *T*_1_, *T*_2_ and diffusion coefficient were performed on an Avance II (Bruker BioSpin GmbH, Rheinstetten, Germany) 9.4 T spectrometer (400 MHz), over the same range of temperatures as above. As for the low-field measurements, *T*_1_ was determined from an inversion-recovery sequence with similar parameters: 16 inversion times, a repetition time of at least 5*T*_1_, two signal averages and no dummy scans. For *T*_2_ measurements, a CPMG sequence was employed but implemented with a last-echo-only sampling scheme. Thus, 16 effective echo-times were sampled using a variable number of echoes to control the time variable. The number and range of echoes varied depending on the sample concentration and temperature, and the echo spacing was 2 ms. Eight averages were acquired, with two dummy scans and a repetition time depending on the sample and temperature.

Diffusion coefficients were measured via a 13-interval bipolar pulsed-gradient sequence [12]. All time parameters were fixed (effective gradient pulse duration *δ* = 5 ms, effective gradient repeat time Δ = 40 ms) while the gradient amplitude was varied for a total of 16 values. The maximum gradient strength used was 0.166 mT mm^−1^. Sixteen averages were acquired with four dummy scans. The repetition time was typically approximately 1 s.

All relaxation and diffusion data were analysed and fitted using Bruker's TopSpin 1.5 software.

## 3. Results

**Figure 1.**
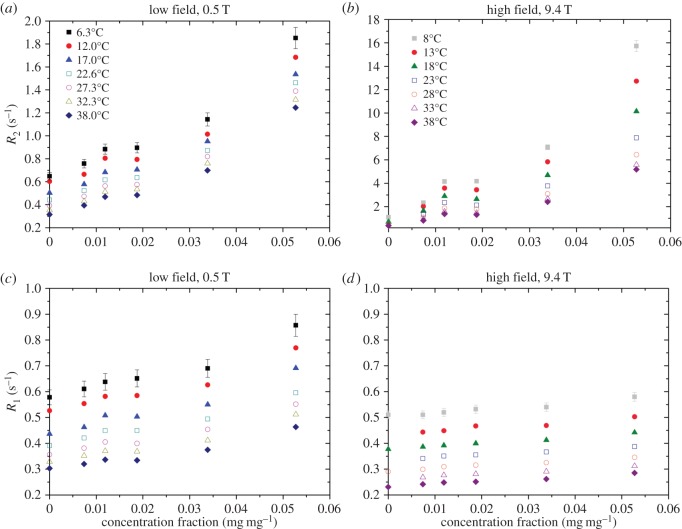
Relaxation measurements as a function of aggrecan solution concentration. (*a*,*c*) Low-field measurement ((*c*), key as in (*a*)) and (*b*,*d*) high-field measurement ((*d*), key as in (*b*)). Temperatures are ±0.5°C for low field, ±0.1°C for high field. The error bars shown are typical for all data (±5% for low field, ±3% for high field) but have not all been displayed for clarity.

Figure 1 shows the collected NMR relaxation rate data for all concentrations and temperatures for low- and high-field measurements. All relaxation rates decrease with temperature and increase with aggrecan concentration. However, there are some clear differences between the four graphs in figure 1; for both field strengths, especially at the higher field, the transverse relaxation rate *R*_2_ is more sensitive to the presence of aggrecan than the longitudinal rate *R*_1_. *R*_2_ increases by over an order of magnitude at high field and by a factor of three to four at the lower field strength, whereas *R*_1_ increases by a factor of less than two at both field strengths (with slightly greater sensitivity at the lower field, contrarily to *R*_2_).

**Figure 2.**
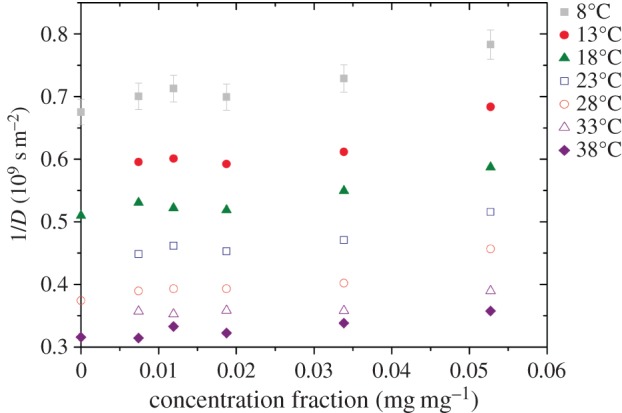
Inverse diffusion coefficients, 1/*D*, as a function of aggrecan solution concentration. Temperatures are ±0.1°C. The error bars shown are typical for all data (±3%) but have not all been displayed for clarity.

Figure 2 shows the results for the diffusion coefficient (plotted as 1/*D*) as a function of concentration and temperature. A weak dependence on concentration is observed which is comparable to the concentration dependence of the high-field *R*_1_ (figure 1*d*).

Using the temperature dependences for both relaxation rates and 1/*D*, an Arrhenius activation energy model [13–15], an equation of the form

**Figure 3.**
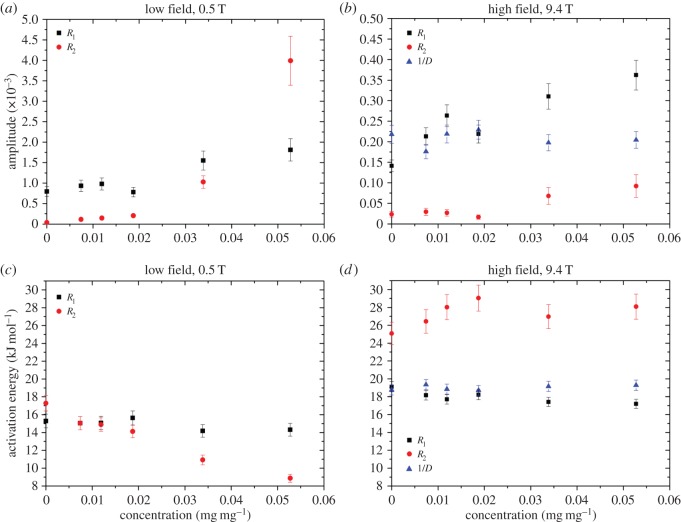
Arrhenius parameters as a function of aggrecan solution concentration. (*a*,*c*) Low-field measurement and (*b*,*d*) high-field measurement. Amplitude is the *A*_0_ term in equation (3.1). For the relaxation rates, the amplitude has units s^−1^. (Note that the amplitude for *R*_2_ in (*a*) has been divided by 10.) For the inverse diffusion coefficient, the amplitude has units 10^9^ s m^−2^.

3.1 $$A=A_{0}e^{E_{A}/RT}$$

can be used to fit the data, where *E*_A_ is the activation energy, *T* is the absolute temperature, *R* is the ideal gas constant, *A*_0_ is an amplitude term and *A* denotes *R*_1_, *R*_2_ or 1/*D*. These results are displayed in figure 3. The amplitudes for the relaxation rates tend to increase with concentration, with the amplitude for *R*_2_ at the lower field strength showing the greatest changes (figure 3*a*). The corresponding activation energies appear to be approximately independent of concentration except for the low-field *R*_2_, which decreases with concentration. The high-field *R*_2_ might also exhibit some concentration dependence at the smaller concentrations. The amplitude and activation energy for 1/*D* appear to be approximately constant.

## 4. Discussion

In the absence of paramagnetic centres, there are several mechanisms which can affect NMR relaxation for spin-1/2 nuclei in macromolecular solutions and biological tissues: dipolar interaction [16,17] between nuclei (intramolecular and intermolecular), chemical exchange [18] and cross-relaxation [17,19–21].

In macromolecular solutions, the water can be considered as existing in several compartments: free (bulk) water, hydrated water and tightly bound (or internal) water [22]. The hydrated water is deemed to be the water which is affected by the presence of a macromolecule or solute but can exchange with the free water. The internal water [23] is that which is bound to the solute in some way that significantly reduces its chance of exchanging with other water compartments (either because it is not connected via a continuous chain of water H-bonds or because it is partly buried in deep narrow cavities). The hydrated and internal water each may exist as a number of distinct compartment types. In the limiting case in which all exchange rates are much greater than the relaxation rates, the relaxation has a single component [24] with an observed relaxation rate which is a weighted sum of the various relaxation rates within the compartments

4.1 $$R_{\mathrm{obs}}=\sum_{i=1}^{n}f_{i}R_{i},$$

where *f_i_* is the fraction of protons in the *i*th compartment with relaxation rate *R*_i_, and where $\sum_{i}^{n}f_{i}=1$ for *n* compartments. If a slow or non-exchanging internal water compartment exists, the relaxing magnetization will not behave as a single component. Typically, such internal water compartments are small fractions of the total water and have short transverse relaxation times and much longer longitudinal relaxation times. In our experiments over the range of parameters used, we saw no compelling evidence for more than one relaxation component.

For hydrated water which is not tightly bound, it is expected that the relaxation rates differ only slightly from the free water values. Correlation times of the rotational and translational motions of the hydrated water will typically be a little longer than those for free water and this will slightly increase the relaxation rates for the hydrated water. In such a scenario, the observed transverse and longitudinal relaxation rates are likely to be similar, as can be seen in the relaxation values for the salt solution in figure 1. However, it is also shown in figure 1 that as the aggrecan concentration increases the transverse relaxation rate *R*_2_ becomes significantly larger than *R*_1_ and this is especially true for the high-field measurements. One mechanism which can explain this behaviour is chemical exchange between sites of different chemical shifts [25].

Protons in water may exchange with labile protons on macromolecules, such as within amine or hydroxyl groups [26]. If we assume that the relevant exchange rates are in the fast-exchange limit (much greater than both the intrinsic relaxation rates and the frequency difference between the chemical environments), expressions for the observed relaxation rates are similar to those in equation (4.1) for the mixing of different water compartments with the exception that for *R*_2_ additional terms are needed which are proportional to the square of the frequency difference [27,28]. The frequency difference is proportional to the field strength and this quadratic field-strength dependence has been demonstrated, for example, in bovine pancreatic trypsin inhibitor solutions by Denisov & Halle [29], and in various protein solutions by Zhong *et al.* [30]. The former authors also showed that the longitudinal relaxation rate decreases with increasing field strength. These basic observations are shown in figure 1; *R*_2_ at high-field is greater than the low-field results, whereas the opposite trend is seen for *R*_1_. This may be interpreted as evidence that chemical exchange between water protons and labile macromolecular protons has played a significant role in determining this relaxation rate.

Cross-relaxation is caused by the dipolar coupling of (hydrated) water protons with macromolecular protons and there is good evidence that cross-relaxation is important in biological tissues [19,20,31,32]. However, it is expected that in dilute solutions of proteins or biopolymers the cross-relaxation rate is negligible compared with the contribution from chemical exchange [33]. Whether the aggrecan solutions used in the experiments presented here can be classed as dilute is uncertain; if cross-relaxation does play a measurable role in the data above then it is expected that the relaxation rate—the contribution from cross-relaxation—is linearly proportional to the concentration of aggrecan and also that the relaxation rate is proportional to the molecular weight of the macromolecule [30,34]. Nonetheless, it is known that the contribution from cross-relaxation to both *R*_1_ and *R*_2_ decreases [35] with field strength, as is typical of the dipolar interaction, and therefore it is likely that chemical exchange is the dominant contribution to the high-field transverse relaxation results.

Approximately equating the aggrecan concentrations *c*_m_ in table 2 with concentrations expressed in units of g cm^−3^, we see that solutions 1–3 are approximately in the physiological range, solution 5 is in the assembly range and solution 4 is more ambiguous, being close to the transition between the two ranges. Referring to the relaxation data of figure 1, especially the transverse relaxation rates, it appears that there is some feature—a plateau, inflection point or a local minimum and maximum—at solutions 3 and 4 (concentrations *c*_m_ = 0.012–0.019). This feature is consistent across many independent measurements and therefore we suggest that this represents a sensitivity in the relaxation rates to a general change in aggrecan-assembly structure, such as self-assembly [3]. If this is the case, self-assembly might affect the accessibility of the aggrecan molecule to water molecules and would thus change the contribution to the relaxation rate from chemical exchange or, indeed, cross-relaxation.

As the concentration increases, if the self-assembly of aggrecan molecules continues to form larger complexes, the molecules will become less mobile. This will lead to more efficient cross-relaxation and could explain the apparent nonlinearity in relaxation rate dependence on concentration that appears to be the case in figure 1. A more detailed investigation of this would be the topic of future work. While it is known that the presence of proteoglycans increases resistance to the flow [36] of water due to the stiff bottlebrush conformation of aggrecan, on the smaller scale the same extended conformation (due to the charged nature of the disaccharides) provides an open structure and allows a relatively free movement of small solvent molecules and solutes [36,37]. The increases in 1/*D* shown in figure 2 are modest and this is probably primarily caused by the obstruction of free diffusion by the aggrecan molecules or assemblies. Evidence for this obstruction effect can be taken from the diffusive activation energy, displayed in figure 3*d*, which shows relative constancy over the range of concentrations, thus indicating that the water structure is not significantly altered by the presence of aggrecan.

The hydrodynamic basis for determining a rotational friction coefficient for spheres rotating within a viscous medium was introduced by Stokes and then later used by Einstein to describe the Brownian motion of particles. The resulting Stokes–Einstein equation can be used to connect the self-diffusion coefficient of the water molecules to the viscosity through

4.2 $$D=\frac{k_{B}T}{6\pi f_{1}\eta r_{h}},$$

where *k*_B_ is Boltzmann's constant, *η* is the local or microviscosity, *r*_h_ is the hydrodynamic radius of the water molecule (1.64×10^−10^ m) and *f*_1_ is a correction term, sometimes known as the microscopic viscosity prefactor [38–40]. For classical Stokes–Einstein behaviour the *f*_1_ parameter, which is dimensionless, has the size unity. There are four key reasons why it can deviate from this value, which are: (i) that the diffusing molecule is anisotropic, reducing *f*_1_ [41]; (ii) that the diffusing molecule is small in size relative to the mixture in which it diffuses, again reducing *f*_1_ (in the limit that the diffusing molecule is large with respect to the solution molecules, *f*_1_ tends to unity) [42]; (iii) that interactions, such as hydrogen bonding, exist within the environment in which it diffuses, making *f*_1_ larger; and (iv) that formation of aggregates increases the effective hydrodynamic radius of the diffusing particle having the phenomenological effect of increasing *f*_1_ [43]. We see that the plots of 1/*D* in figure 2 are (assuming *r*_h_ and *f*_1_ are constant) proportional to the microviscosity.

Clearly, *η* is not strongly dependent on the concentration. If we further assume that the relaxation rate *R*_1_ is dominated by the rotational correlation time *τ*_r_, in the limit that $\omega_{0}\tau_{r}\ll 1,\;$ where *ω*_0_ is 2*π* times the resonance frequency (400 MHz at 9.4 T), then

4.3 $$R_{1}\approx 5\;K\tau_{r},$$

where *K* ≈ 1.392 × 10^10^ s^−2^ for protons in water. Using a similar relation to the Stokes–Einstein equation for rotational diffusion, the rotational correlation time can be expressed as

4.4 $$\tau_{r}=\frac{4\pi f_{2}\eta r_{h}^{3}}{3k_{B}T},$$

where *f*_2_ is the microscopic viscosity prefactor [43], which is not necessarily equal to the prefactor *f*_1_ and can also vary in the same manner for the same key reasons described above, in equation (4.2). Taking the product *DR*_1_ and using the above relations, we find

4.5 $$DR_{1}=\frac{10}{9}\frac{f_{2}}{f_{1}}Kr_{h}^{2}.$$

Using the values for *K* and the hydrodynamic radius given above,

4.6 $$DR_{1}\approx 0.42\times 10^{-9}\frac{f_{2}}{f_{1}}\;m^{2}\;s^{-2}.$$

**Figure 4.**
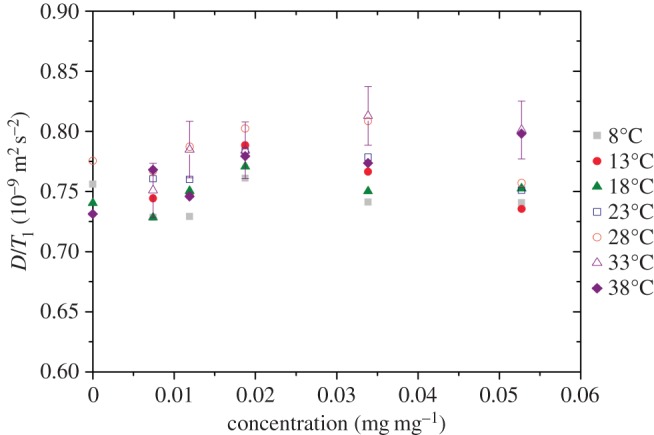
Ratio of the diffusion coefficient to the high-field relaxation time *T*_1_ as a function of aggrecan solution concentration. Temperatures are ±0.1°C. The error bars shown are typical for all data (±3%) but have not all been displayed for clarity.

Using the experimental data for *R*_1_ in figure 1*d* and the diffusion data of figure 2, the product *DR*_1_ (= *D*/*T*_1_) is plotted in figure 4. For all temperatures and all concentrations, the average value is 0.77 ± 0.04×10^−9^ m^2^ s^−2^. From this, we can determine the ratio *f*_2_/*f*_1_ (in equation (4.6)) to be 1.8 ± 0.1. We can compare this ratio to that of pure water. Using values of *D* = 2.023×10^−9^ m^2^ s^−1^, *η* = 1.0019 mPa s, *T*_1_ = 3.15 s, at a temperature of 20°C, we calculate a ratio *f*_2_/*f*_1_ = 1.54. This value is close to the ratio calculated for the aggrecan solutions. This, and the fact that *f*_2_/*f*_1_ is approximately constant, suggests that both *R*_1_ and diffusion are essentially functions of the local microviscosity with the dominant mechanism for the former being rotational motion.

The activation energy data for both diffusion (figure 3*d*) and amplitude (figure 3*b*) are independent of aggrecan concentration. As above, we infer this to mean that the aggrecan molecules were only acting as barriers to diffusion and the structure of the water between the macromolecules was not significantly affected. The activation energy for *R*_1_ (both high and low field) is also approximately independent of concentration. The amplitude, however, is not constant, instead showing a roughly linear increase with concentration at both high- and low-field strengths. The amplitude term represents the relaxation time or diffusion at infinite temperature and so is related to the configurational degrees of freedom available to the system for diffusion or relaxation and, in turn, the entropy of the system. The increase in amplitude thus suggests the increase in concentration results in a reduction in the number of pathways for rotation due to configurational restrictions [14].

The activation energy for *R*_2_, however, shows more variation; at low field, the activation energy for *R*_2_ showed a linear decrease with increasing concentration, yet at high field, this same parameter showed a linear increase at lower concentrations, but then flattened off at higher concentrations. In general, the Arrhenius parameters for *R*_2_ are more complicated than for *R*_1_ or diffusion. For example, Fung & McGaughy [44] measured relaxation rates (at a field strength of approx. 0.22 T) and their activation energies in muscle tissue. For protons, they measured 9.1 and −1.3 kJ mol^−1^, respectively, for *R*_1_ and *R*_2_. They attributed these low values (especially for *R*_2_) to chemical exchange between water protons and labile macromolecular protons and provided evidence for this conclusion by comparing activation energies for oxygen-17 (which does not undergo chemical exchange [45,46]). The activation energies they found for ^17^O were 18 and 14 kJ mol^−1^, respectively, for *R*_1_ and *R*_2_. Note that the ^17^O activation energy for *R*_1_ is very close to our high-field measurements for *R*_1_ and diffusion. The negative value quoted above for the *R*_2_ activation energy of protons suggests that chemical exchange may be important even at low fields for water in muscle and thus both our high- and low-field measurements are likely to be complicated by this mechanism.

The Arrenhius amplitude term for *R*_2_ also shows variation; a slow linear increase with concentration at high field and an exponential increase with concentration for the low-field measurements. As with the amplitude term for *R*_1_, this is also believed to be due to the entropy in the system for relaxation [14].

An important aspect, which was not considered during this work, particularly in the context of cartilage response to compressive loading, is the effect of osmolarity. In this work, we used a fixed concentration of salt solution while investigating the effects of the aggrecan concentration but it is known that calcium ions, in particular, play an important role [47–50] in the structure and function of cartilage and its constituents.

## 5. Conclusion

We have measured proton transverse and longitudinal relaxation rates and diffusion coefficients as a function of aggrecan solution concentration and temperature. We compared the relaxation rates at low field (0.5 T) and high field (9.4 T). We found that the transverse relaxation rate *R*_2_ was the most sensitive to the aggrecan concentration, particularly so at the higher field strength where chemical exchange is likely to be the dominant mechanism. The longitudinal relaxation rate *R*_1_ was much less sensitive to aggrecan concentration and also exhibited the opposite trend with regard to field strength; that is, *R*_1_ was more sensitive to aggrecan concentration at the *lower* field.

The inverse diffusion coefficient was found to be approximately proportional to the high-field *R*_1_ such that the product *DR*_1_ was constant for all temperatures and concentrations. This observation suggests that both the *R*_1_ process (high field) and diffusion are controlled by the local microviscosity.

Activation energies for *R*_1_ and diffusion appeared to be approximately independent of concentration which suggests that the presence of aggrecan does not significantly alter the water structure away from the aggrecan molecules. The activation energy for *R*_2_, however, showed a more complicated behaviour with concentration and field strength, again, presumably due to the effects of chemical exchange.

While future work is required to confirm these conclusions, we believe this approach offers promise in strengthening our understanding of magnetic resonance methods as applied to articular cartilage.

## Data accessibility

Data from: an NMR Study of Water in Aggrecan Solutions. Dryad Digital Repository. http://dx.doi.org/10.5061/dryad.0dg47.
